# Exploring Connections Between Weight‐Loss Medications and Thyroid Cancer: A Look at the FDA Adverse Event Reporting System Database

**DOI:** 10.1002/edm2.70038

**Published:** 2025-03-08

**Authors:** Christophe Abi Zeid Daou, Omar Aboul Hosn, Lana Ghzayel, Marc Mourad

**Affiliations:** ^1^ Department of Otolaryngology and Head and Neck Surgery American University of Beirut Medical Center Beirut Lebanon

**Keywords:** diabetes, thyroid cancer, weight loss

## Abstract

**Aims:**

GLP‐1 receptor agonists, such as semaglutide (Ozempic) and tirzepatide (Monjaro), have gained significant popularity for obesity management, but concerns have arisen about their potential link to thyroid cancer. This study investigates the association between thyroid cancer and weight‐loss medications.

**Materials and Methods:**

A disproportionality analysis was conducted using data from the FDA Adverse Event Reporting System (FAERS) from 2004 to Q1 2024. Reporting odds ratios (RORs) were used to identify associations between thyroid cancer and weight‐loss drugs, including anti‐diabetic medications.

**Results:**

Significant positive associations with thyroid cancer were found for GLP‐1 receptor agonists: semaglutide (ROR = 7.61, 95% CI: 6.37–9.08), dulaglutide (ROR = 3.59, 95% CI: 3.03–4.27), liraglutide (ROR = 15.59, 95% CI: 13.94–17.44) and tirzepatide (ROR = 2.09, 95% CI: 1.51–2.89). A weak inverse association was observed for metformin (ROR = 0.58, 95% CI: 0.36–0.93). No significant associations were found for other drugs, such as topiramate, dapagliflozin and insulin glargine.

**Conclusion:**

The study, based on data from the FAERS database, suggests a potential association between GLP‐1 receptor agonists and an increased thyroid cancer risk. These findings underscore the importance of further research and continuous safety monitoring when prescribing these medications for obesity management.

## Graphical abstract

This study explores the potential link between GLP‐1 receptor agonists and thyroid cancer using data from the FDA Adverse Event Reporting System (FAERS). Disproportionality analysis reveals significant positive associations for GLP‐1 receptor agonists, underscoring the need for further research and ongoing safety monitoring in obesity management.

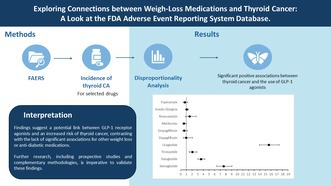

## 1 Introduction

There has been a noticeable surge in the adoption of weight loss medications in a proactive approach to obesity management and its associated health risks [1, 2]. Among the pharmaceutical options that have garnered significant attention are Orlistat, a lipase inhibitor that reduces fat absorption in the gastrointestinal tract; Naltrexone/Bupropion, a combination therapy targeting both the opioid and dopamine/norepinephrine pathways to suppress appetite and increase metabolism [3]; Liraglutide and Semaglutide (Ozempic), glucagon‐like peptide‐1 receptor agonists originally used in diabetes management but repurposed for weight loss due to their appetite‐suppressing effects [4]; and Phentermine/Topiramate, a combination of a sympathomimetic amine and an antiepileptic drug that reduces appetite and increases satiety [5].

Over the past few years, GLP‐1 receptor agonists have gained global popularity, namely Ozempic (Semaglutide) and Mounjaro (Tirzepatide). With the former emerging as one of the most prescribed medications, with over 9 million prescriptions recorded in the final quarter of 2022 [1, 5].

Prescriptions for GLP‐1 receptor agonists have surged by over 300% since 2020 [6]. This significant increase underscores the importance of vigilant monitoring for adverse events and potential sequelae associated with their use. Healthcare providers must carefully consider patient‐specific factors, such as underlying conditions and concurrent medication use, to ensure safe and effective treatment outcomes. GLP‐1 receptor agonists are primarily excreted by the kidneys, and their pharmacokinetics can be influenced by renal function. In cases of severe renal impairment, certain GLP‐1RAs may require dosage adjustments or careful monitoring to avoid adverse effects such as accumulation or toxicity. For example, agents like exenatide are contraindicated in severe renal impairment (eGFR < 30 mL/min/1.73m^2^), while others like dulaglutide and semaglutide are not as reliant on renal clearance, making them potentially safer options in such patients. Dose adjustments are unnecessary in patients with liver dysfunction [7, 8]. Although concerns have been raised regarding potential interactions with the cytochrome P450 system as well as the effects of delayed gastric emptying induced by these medications, clinical studies have shown that such interactions generally do not require dose adjustments for commonly co‐administered drugs. Medications such as warfarin, oral contraceptives, acetaminophen and statins have been specifically evaluated in this context. Results indicate that GLP‐1RAs do not significantly alter the pharmacokinetics or efficacy of these agents, making them compatible for concurrent use without modification of dosage [9]. Extensive meta‐analyses have highlighted the efficacy and safety of GLP‐1RAs in achieving glycemic control, enhancing quality of life and significantly reducing major cardiovascular events in both diabetic and non‐diabetic individuals with obesity. Adverse effects are predominantly mild and transient, with nausea being the most commonly reported [10, 11]. Despite regulatory concerns about psychiatric effects, such as suicidal ideation, current evidence indicates no significant increase in the incidence of depression, anxiety, or suicidal behaviour among GLP‐1RA users. Semaglutide, in particular, has been associated with rare psychiatric events, including anxiety and sleep disturbances, yet these occurrences have not been shown to have clinically significant impacts on depressive symptoms or suicidal ideation [12, 13]. This evidence supports the safety of GLP‐1RAs in polypharmacy settings, though continued monitoring for individual variability remains prudent.

Despite their effectiveness in treating type 2 diabetes and obesity, concerns remain about the potential link between GLP‐1RAs and thyroid cancer. Preclinical studies suggest that GLP‐1 receptor activation promotes C‐cell proliferation and increases the risk of thyroid cancer, including medullary thyroid carcinoma (MTC), in rodents, likely due to higher GLP‐1 receptor expression in thyroid cells [14, 15]. However, human data remain inconclusive, as GLP‐1 receptors are minimally expressed in human thyroid cells [16]. Clinical findings vary, with some studies reporting no significant changes in serum calcitonin or thyroid malignancies after long‐term liraglutide use [17]. Others suggest an increased risk of thyroid cancer, particularly MTC, with 1–3 years of GLP‐1RA use. Pharmacovigilance data also point to a potential association with thyroid cancer, particularly with liraglutide and exenatide, emphasising the need for further research to assess the effects of chronic GLP‐1RA exposure on thyroid health, especially in individuals with premalignant lesions or occult nodules [18, 19].

These findings underscore the importance of comprehensive risk assessment and regular surveillance when prescribing weight loss medications, highlighting the need for further research to elucidate their safety profiles and optimise their clinical utility in combating the global epidemic of obesity.

## 2 Materials and Methods

### 2.1 Data Source and Extraction

An Institutional Review Board approval was not needed for this type of study as the FDA Adverse Event Reporting System (FAERS) database contains data on spontaneously reported adverse events (AEs) and medication errors, which is publicly available. Considering the drug's market introduction timeline, we conducted a disproportionality analysis of FAERS spanning from 2004 to the first quarter of 2024, utilising odds ratios to identify potential associations between thyroid cancer and weight loss‐inducing drugs. Disproportionality analysis serves as a validated method for detecting significant links between drugs and AEs [20]. Our investigation encompassed anti‐diabetic medications known to induce weight loss, such as semaglutide, dulaglutide, liraglutide, tirzepatide, empagliflozin, dapagliflozin and canagliflozin. We also included weight‐neutral diabetic medications including metformin, sitagliptin, linagliptin, alogliptin, vildagliptin, saxagliptin and pioglitazone. Other weight loss‐inducing drugs like orlistat, bupropion, topiramate, phentermine and naltrexone were alsoincluded.

A meta‐analysis has demonstrated that diabetes mellitus is associated with an increased risk of thyroid cancer, with patients with type 2 diabetes having a 1.34‐fold higher risk compared to non‐diabetic individuals (95% CI, 1.17–1.53) [21]. To address this inherent risk and reduce potential confounding, we included weight‐neutral medications frequently prescribed to diabetic patients, such as insulin glargine, sitagliptin, rosuvastatin, metformin and glyburide, in our analysis. To capture reports where these medications were listed as concomitant medications rather than solely as primary or secondary suspects for the AE of interest, no filter was applied based on the role of the drug. Drugs associated with fewer than five drug‐event pairs (i.e., drug‐thyroid cancer combinations) were excluded from the analysis. The threshold of “≤ 5” drug‐event pairs was chosen to improve the reliability and validity of the disproportionality analysis by reducing the risk of spurious associations due to small sample sizes. In pharmacovigilance studies, using thresholds like this helps prevent the calculation of unstable or misleading signals where low reporting counts may result in inflated or misleading ROR [22, 23]. Adverse events included: thyroid cancer, thyroid neoplasm, recurrent thyroid cancer, papillary thyroid cancer, follicular thyroid cancer, medullary thyroid cancer and anaplastic thyroid cancer. The data manipulation process was performed using Microsoft Excel 2021 and IBM SPSS Statistics for Windows, version 28 (IBM Corp., Armonk, NY, USA), which together facilitated comprehensive data processing and analysis.

### 2.2 Data Analysis

Disproportionality analysis is a widely used pharmacovigilance tool that helps detect potential associations between drugs and adverse events by identifying disproportionately reported events compared to others in a database. It is particularly valuable for signal detection, highlighting adverse events that occur more frequently with a specific drug and serves as an efficient method for analysing large datasets like those from the FAERS. It also acts as an early warning system for rare or unexpected events. These findings guide regulatory actions, prioritise further research and enhance post‐marketing drug safety monitoring. Disproportionality was quantified using the reporting odds ratio (ROR) and its corresponding 95% confidence interval (CI), where ROR represents the odds of thyroid cancer occurrence with the drug of interest compared to the odds with all other drugs in the database. The ROR was calculated using 2 × 2 contingency tables for analysis. To note that ROR does not measure absolute risk or predict causal relationships. Instead, it highlights disproportionality in reporting, which warrants further investigation in a specific direction.

## 3 Results

### 3.1 Population Characteristics

**FIGURE 1:**
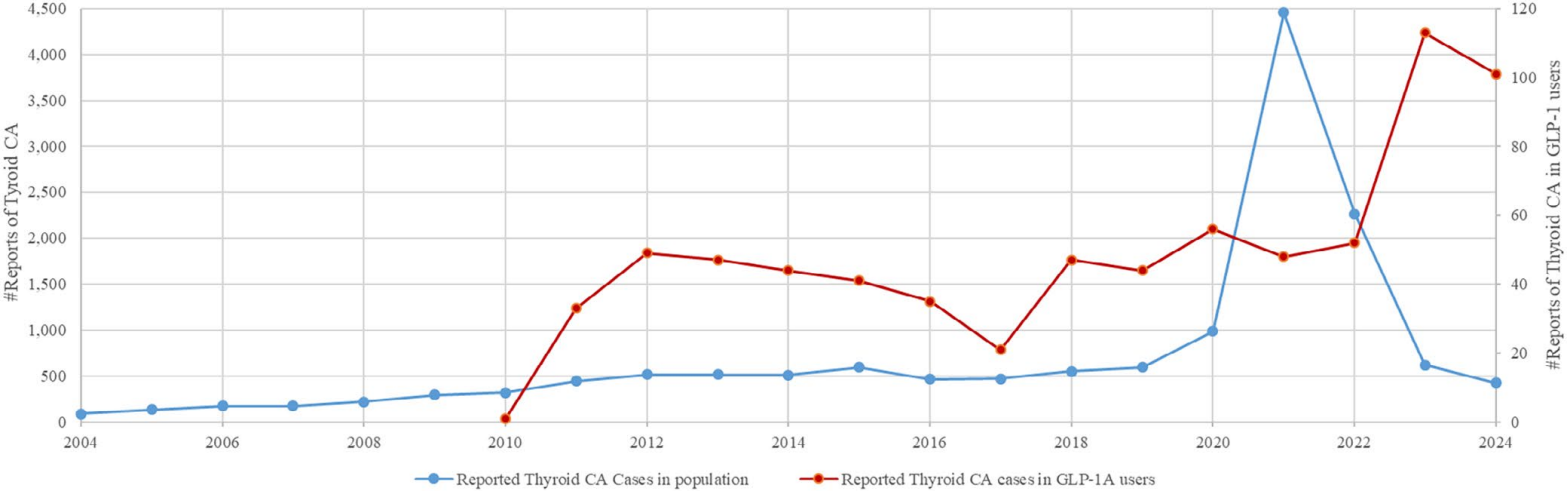
Yearly trend of reported thyroid cancers associated with GLP‐1 receptor agonists.

From 2004 to 2024, a total of 14,370 adverse events (AEs) related to drug‐induced thyroid cancer were reported. The year 2021 recorded the highest number of reported cases, exceeding 4000, as illustrated in Figure 1. The clinical characteristics of these reports are summarised in Table 1. The majority of patients were adults aged 18 to 64, with a predominance of female cases. The reported outcomes were mostly generic (e.g., “cancer” or “neoplasm”) and less frequently specified by pathology. Among classified cases, papillary thyroid cancer accounted for 9.1%, followed by medullary thyroid cancer at 1.5%. Geographically, most reports originated from the United States (44.85%), with additional reports from Canada (3.4%), Japan (1.1%) and Germany (0.81%).

**TABLE 1:** Characteristics of reported drug‐induced thyroid cancer.

Characteristics	Percentage of AE (%)	Percentage of AE in GLP‐1A users (%)
Sex		
Female	63	60.4
Male	23.6	28.5
Non‐Specified	13.4	11.1
Age		
0–11 years	0.4	0
12–17 years	0.6	0.1
18–64 years	42.8	37.3
65–85 years	11.1	12.5
Non‐Specified	44.9	50.1
Report Classification		
Thyroid Cancer	77	43
Thyroid Neoplasm	11	16.8
Papillary Thyroid Cancer	9.1	22.4
Medullary Thyroid Cancer	1.5	14.3
Follicular Thyroid Cancer	0.8	2.9
Other	0.6	0.6
Reporting Country		
Unites States of America	44.85	66.9
Canada	3.40	3.3
Japan	1.10	0.2
Germany	0.81	1.4
France	0.76	1.1
Italy	0.70	0.2
Other	48.38	26.9

### 3.2 Disproportionality Analysis

The number of reports for thyroid cancer as an event versus reports of other events for each weight of the studied drugs is shown in Table 2. The following medications were not included in the analysis due to an inadequate number of reports (i.e., ≤ 5): phentermine, naltrexone, orlistat, glyburide, bupropion, sitagliptin, alogliptin, vildagliptin, saxagliptin and pioglitazone.

**TABLE 2:** Number of reports of thyroid cancer as an adverse event versus reports of other adverse events for each drug of interest based on the data from the FDA Adverse Event Reporting System database.

Drug	Class	Number of reports with thyroid cancer as an event	Number of reports with events other than thyroid cancer
Semaglutide	GLP‐1 receptor agonist	124	29,401
Dulaglutide	GLP‐1 receptor agonist	131	65,684
Liraglutide	GLP‐1 receptor agonist	316	36,562
Tirzepatide	Dual GIP and GLP‐1 receptor agonist	37	31,928
Empagliflozin	SGLT‐2 inhibitor	11	30,778
Dapagliflozin	SGLT‐2 inhibitor	7	12,714
Metformin	Biguanide	17	53,172
Phentermine	Sympathomimetic	0	1427
Naltrexone	Opioid receptor antagonist	2	24,375
Orlistat	Lipase inhibitor	4	23,841
Topiramate	Sodium channel blocker and glutamate inhibitor	14	34,652
Bupropion	Norepinephrine‐dopamine reuptake inhibitor	0	17,692
Rosuvastatin	HMG‐CoA reductase inhibitors	12	13,890
Glyburide	Sulfonylurea‐ insulin secretagogue	1	4617
Insulin Glargine	Insulin	63	10,4510
All drugs	Any	14,370	25,925,016

**FIGURE 2:**
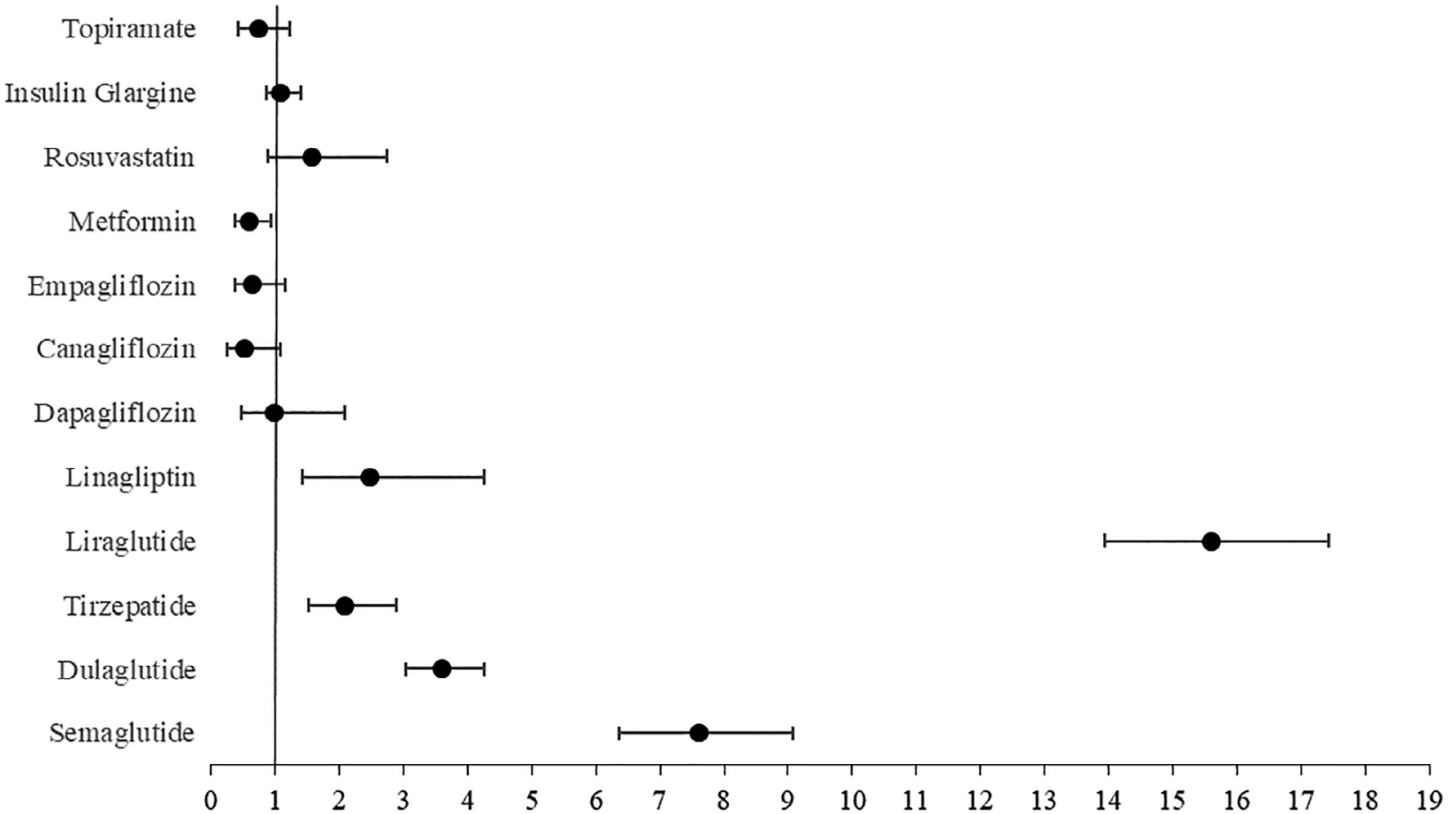
Reporting odds ratios (RORs) for thyroid cancer associated with weight loss medications.

The results of the disproportionality analysis are presented in Figure 2. Positive associations (*p* < 0.0001) were found between thyroid cancer development and the following anti‐diabetic weight loss‐inducing drugs: semaglutide (ROR = 7.61; 95% CI: 6.37–9.08), dulaglutide (ROR = 3.59; 95% CI: 3.03–4.27), liraglutide (ROR = 15.59; 95% CI: 13.94–17.44) and tirzepatide (ROR = 2.09; 95% CI: 1.51–2.89). There was also a positive association between thyroid cancer development and linagliptin (ROR = 2.47; 95% CI: 1.43–4.26). We also found a weak inverse association (*p* = 0.02) with metformin (ROR = 0.58; 95% CI: 0.36–0.93).

The yearly trend of reported thyroid cancers associated with GLP‐1 receptor agonists shows a steady rate of approximately 40–50 cases per year from 2012 to 2022, as illustrated in Figure 1. However, a significant increase in reports was observed in 2023 and 2024. These reports were predominantly among females (60%) and adults aged 18–64 (37%), reflecting a distribution consistent with the demographic trends observed in the studied population (Table 1).

No significant associations were found for other weight loss‐inducing drugs such as topiramate (ROR = 0.73; 95% CI: 0.43–1.23). Also, no significant associations were found for other diabetic medications such as dapagliflozin (ROR = 0.99; 95% CI: 0.47–2.08), empagliflozin (ROR = 0.65; 95% CI: 0.36–1.16), canagliflozin (ROR = 0.52; 95% CI: 0.25–1.08), insulin glargine (ROR = 1.08; 95% CI: 0.85–1.39) or even with rosuvastatin (ROR = 1.56; 95% CI: 0.89–2.75).

The results remained consistent when stratifying by age or sex. Regarding thyroid cancer types, the majority of reports were generic and not classified by specific pathology (98%). Among cases where the type was reported, papillary thyroid cancer was the most common (51.2%), followed by medullary thyroid cancer (36%), reflecting general reporting trends. Analysis of individual drugs revealed that all showed higher numbers of papillary thyroid cancer cases, except for semaglutide and dulaglutide, which exhibited comparable rates of papillary and medullary thyroid cancers (Table 3).

**TABLE 3:** Proportion of different histological subtypes of thyroid cancer, as reported by FAERS.

Drug	Medullary	Papillary	Metastatic	Other
Semaglutide	23.8%	23.1%	2.7%	50.4%
Dulaglutide	13.1%	13.1%	1.5%	72.3%
Liraglutide	11.8%	25.5%	1.2%	61.5%
Tirzepatide	13.7%	23.5%	—	62.8%
Empagliflozin	27.3%	9%	—	63.7%
Dapagliflozin	—	14.3%	—	85.7%
Metformin	—	11.8%	—	88.2%
Topiramate	—	13.3%	—	86.7%
Rosuvastatin	—	—	—	100%
Insulin Glargine	9.2%	3%	—	87.8%

*Note:* Other includes anaplastic, poorly differentiated cancers and unspecified pathology.

## 4 Discussion

We found a positive association between GLP‐1 receptor agonists and tirzepatide and the incidence of thyroid cancer. These findings suggest a potential long‐term side effect of these medications, especially as the same association was not found with other anti‐diabetic or weight loss medications.

Clinical evidence regarding an association between GLP‐1 receptor agonists (GLP‐1RAs) and thyroid cancer remains inconclusive.

Despite the paucity of data, studies in the literature have not found an association between thyroid cancer and GLP‐1RAs. Some reports looking at semaglutide found the incidence of thyroid cancer to be low, representing only 1% of the study population [24]. In a study looking at the risk of thyroid cancer with semaglutide, liraglutide, exenatide and dulaglutide, only 86 cases of thyroid cancer were identified among 69,909 patients (0.1%). A Scandinavian cohort study looking at 145,410 patients using GLP1‐agonists also found no significant increased risk of thyroid cancer over a mean follow‐up of 3.9 years (HR = 0.90, 95% CI 0.58 to 1.38) [25]. Moreover, cardiovascular outcome trials for GLP‐1 receptor agonists demonstrated stable calcitonin levels in patients over 3 years of drug exposure, with no observed increase in the risk of newly diagnosed medullary thyroid carcinomas [26]. Furthermore, a meta‐analysis conducted by Hu et al. in 2022 found that GLP‐1RA had no significant effects on the occurrence of thyroid cancer (RR 1.30, 95% CI 0.86–1.97), thyroid masses (RR 1.17, 95% CI 0.43–3.20) and goitre (RR 1.17, 95% CI 0.74–1.86) [27].

Some studies, on the other hand, found an increased risk of cancer in these patients with long‐term use. Namely, the study by Bezin et al. found an increased risk for developing medullary thyroid cancer in patients using GLP‐1 agonists for 1–3 years, with an adjusted HR of 1.78 (95% CI 1.04–3.05) and an adjusted HR of 1.58 (95% CI 1.27–1.95) for all thyroid cancers [28].

A meta‐analysis by Silverii et al. also found a significant increased risk of thyroid cancer in these patients with an odds ratio of 1.52 ([95% CI 1.01, 2.29]; *p* = 0.04, I2 = 0%), with a 5‐year number needed to harm of 1349. The results were not significant when then stratified by cancer type (papillary or medullary) [16].

A recent study utilising the French National Health Cancer Data System found that using GLP‐1RAs for 1 to 3 years was associated with an increased risk of all thyroid cancers (adjusted HR, 1.78; 95% CI 1.04–3.05) [29]. These findings align with current package warnings for GLP‐1RAs, which highlight their contraindication in patients with multiple endocrine neoplasia syndrome type 2 (MEN2) and emphasise counselling patients about the potential risk of medullary thyroid cancer (MTC) and symptoms of thyroid tumors [30].

However, definitive evidence linking GLP‐1RAs to increased cancer incidence remains elusive, primarily due to methodological challenges. Lag‐time bias and the relatively short duration of clinical trials limit their ability to capture long‐term cancer outcomes. Extended follow‐up periods and larger‐scale observational studies are needed to clarify this potential association further.

The relationship between GLP‐1 receptor agonists (GLP‐1 RAs) and thyroid cancer risk is complex, with varying results reported in the literature. These inconsistencies can be attributed to several factors, including differences in study design and population, as well as possible variations in GLP‐1 RA formulations. Importantly, there are significant confounding factors that complicate the interpretation of these studies.

Obesity is a major risk factor for thyroid cancer, independent of GLP‐1 RA use. Studies have shown that overweight individuals face a 25% increased risk of developing thyroid cancer, while obese individuals have a 55% increased risk compared to their normal‐weight counterparts [31]. Additionally, for every 5‐unit increase in BMI, as an independent variable, the risk of thyroid cancer was shown to increase by 30% (RR = 1.41 in men and RR = 1.25 in women) [31]. Diabetes is another independent risk factor for thyroid cancer. A 10‐year prospective study reported a 25% increased risk of thyroid cancer in patients with diabetes, with an even higher risk observed in females (HR = 1.46, 95% CI: 1.01–2.10) [11]. However, it is worth noting that this study did not document GLP‐1 RA intake. Our study found no disproportionality in thyroid cancer risk with other weight loss or anti‐diabetic medications. It is crucial to emphasise that the increased thyroid cancer risk associated with obesity and diabetes exists independently of GLP‐1 RA use. This complicates the interpretation of studies examining the relationship between GLP‐1 RAs and thyroid cancer, as these underlying conditions are common in patients prescribed GLP‐1 RAs. Future research should carefully control for these confounding factors to isolate the potential effects of GLP‐1 RAs on thyroid cancer risk.

The role of GLP‐1RAs in cancer pathogenesis remains complex and inconclusive, with evidence neither firmly establishing nor entirely refuting their involvement in specific cancer types. A systematic review found no definitive causal association between GLP‐1RAs and pancreatic cancer, reporting odds ratios of 0.93 (95% CI: 0.65–1.34, *p* = 0.71) and 0.94 (95% CI: 0.52–1.70, *p* = 0.84) in comparison to control arms [32]. Furthermore, a target trial emulation by Wang et al. demonstrated a significantly reduced risk of pancreatic cancer incidence in patients treated with GLP‐1RAs compared to other anti‐diabetic medications (HR = 0.42–0.82) [33]. Studies also looked at the effect on prostate cancer; some research indicates that GLP‐1RAs may influence pathways related to cellular proliferation and apoptosis. A meta‐analysis by Sharma et al. looking at five studies from the literature revealed an RR of 0.72 (95% CI: 0.610 to 0.832), indicating a statistically significant 28% reduction in prostate cancer risk associated with GLP‐1RA use compared to placebo or other antidiabetic drugs [34]. Similarly, these medications have shown a protective effect in the incidence of colorectal cancer. Research indicates that liraglutide can reduce cell proliferation, migration and invasion while promoting apoptosis in colorectal cancer cells [35]. In relation to oesophageal and gastric cancers, the use of GLP‐1RAs in patients with T2DM does not seem to significantly increase the risk of gastric or oesophageal cancer; however, further studies are still needed [36].

Regarding metformin, our findings suggest a potential protective role against thyroid cancer, as indicated by significantly fewer adverse event reports associated with this medication (ROR = 0.58; 95% CI: 0.36–0.93). This aligns with evidence from a meta‐analysis that demonstrated a statistically significant reduction in thyroid cancer risk with metformin use (pooled OR = 0.68; 95% CI: 0.50–0.91; *p* = 0.011), with a particularly pronounced protective effect in Eastern populations, showing a 45% risk reduction [37]. Further studies have observed that long‐term metformin use—beyond 1633 days and with mean doses of 868,169 mg—resulted in a 31% reduction in thyroid cancer risk [38]. These anticancer effects are thought to be mediated through interference with insulin/IGF signalling and modulation of the AMPK/mTOR pathway, which inhibits unchecked cell cycle progression, induces apoptosis in thyroid cancer cells, and reduces colony formation and migration [37].

As for linagliptin, evidence regarding its effect on thyroid cancer progression remains inconclusive due to limited studies. Some pharmacovigilance analyses have suggested a potential association with other malignancies, such as liver cancer (ROR = 0.84; 95% CI: 0.65–1.07) [39], though this association has not been consistently observed. For instance, a population‐based cohort study by Bea et al. found no significant increase in thyroid cancer risk (HR = 0.95; 95% CI: 0.79–1.14) when comparing DPP‐4 inhibitors, such as linagliptin, to SGLT2 inhibitors [40].

This study has its limitations and does not offer conclusive evidence of a causal relationship between product exposure and reported events, as adverse events may be linked to the underlying medical condition, interactions with other medications, or unrelated factors. Furthermore, the absence of a control group makes it difficult to distinguish between true associations and coincidental findings. As a voluntary reporting system, FAERS inherently overlooks certain adverse events due to underreporting or inconsistent reporting, resulting in an incomplete representation of data. Additionally, the database does not provide a denominator, such as the total number of prescriptions or patient exposures, which prevents the calculation of accurate incidence rates. The database often contains duplications, and some submissions lack sufficient detail to draw meaningful conclusions. The data in these reports reflect the observations and opinions of reporters rather than verified medical evidence; a report does not confirm the accuracy of the included information, nor does it represent an admission of causality by the reporter. These inherent issues preclude the use of FAERS data to calculate the rates of adverse events; rather, a disproportionality analysis is used.

Another critical limitation is the potential for reporting bias, as FAERS relies on spontaneous reporting, which can lead to overrepresentation or underrepresentation of certain adverse events. This bias may be influenced by public or clinician perceptions regarding a possible association between a drug and an adverse event rather than objective findings.

Finally, the inability to adjust for clinical characteristics or evaluate synergistic effects due to the database's structure poses additional challenges. These limitations underscore the need for cautious interpretation of FAERS data and highlight the importance of conducting further research, ideally through robust controlled studies, to validate and expand upon these findings.

## 5 Conclusion

In conclusion, this study, based on data from the FAERS database, suggests a potential association between GLP‐1 receptor agonists and an increased risk of thyroid cancer. However, it is important to note that these findings are limited by the nature of the FAERS database, which relies on voluntary and spontaneous reporting, lacks a control group, and cannot establish causation. These limitations highlight the need for further Validation of these findings through complementary methodologies and prospective studies to gain a robust understanding of the safety profile of GLP‐1 receptor agonists.

These results underscore the importance of guiding patient counselling about the potential risks of these medications while encouraging further research to explore the broader implications of anti‐diabetic medications with weight‐loss properties. Comprehensive safety evaluations are essential to ensure informed decision‐making in clinical practice.

## Author Contributions

C.A.Z.D. was involved in the inception of the idea, data collection and analysis and manuscript writing and reviewer comment incorporation. O.A.H. was involved in manuscript rewriting and reviewer comment incorporation. L.G. was involved in reviewing, proofing and submitting the manuscript. M.M. was involved in manuscript editing and project supervision.

## Conflicts of Interest

The authors declare no conflicts of interest.

## Data Availability

The datasets generated during and/or analysed during the current study are available from the corresponding author upon reasonable request.
